# Exploring innovation in challenging contexts: The experiences of ethnic minority restaurant owners during COVID-19

**DOI:** 10.1177/14657503211051219

**Published:** 2023-02

**Authors:** Tayo Korede, Abdullah Al Mamun, Paul Lassalle, Andreas Giazitzoglu

**Affiliations:** Lancashire School of Business & Enterprise, 6723 University of Central Lancashire, UK; 5994 Newcastle University Business School, UK; 150880 Hunter Centre for Entrepreneurship, Strathclyde Business School, University of Strathclyde, UK; 5994 Newcastle University Business School, UK

**Keywords:** Innovation adoption, survival, technology, ethnic minority restaurant owners, COVID-19

## Abstract

This article explores how Bangladeshi immigrants who run and own restaurants in the West Midlands of England (UK) participated in forms of innovation in response to the challenges created by COVID-19. Contributing to debates on innovation and diversification in the ethnic minority entrepreneurship literature, we explore through qualitative interview data how restaurant owners innovatively engaged with particular resources to secure their survival and longer-term futures in localised economies. This form of innovation is significant as it occurs among a population of entrepreneurs who have traditionally been portrayed as reluctant to innovate and embrace change. Our study therefore explores how a long-held culturally rooted reluctance to innovate intersects with a contemporary need to innovate for a demographic responding to the crisis. We theorise the form of innovation we identify as situated between a forced bricolage and a neoclassical approach to innovation.

## Introduction

Curry restaurants have been an integral part of the British culinary landscape since 1940 (Narayan, 1995). Today, there are approximately 12,000 curry restaurants across the UK, which employ 100,000 people and have annual sales of £4.2 billion (Financial Times, 2016). However, the COVID-19 pandemic threatened the survival of many restaurants in the UK. In turn, a particular need has emerged for curry restaurants to respond to the challenges they face, through forms of innovation. However, this innovation does not occur in a vacuum, but against a particular backdrop that sees cultural context, innovation and crisis intersect.

Based on rich interview data, we draw on restaurant owners’ qualitative experiences of the pandemic, its impact on their ventures and how they responded to the crisis using particular *forms* of innovation. Via our empirics, building on a growing collection of work looking at business development, diversification and innovation adoption of ethnic minority ventures (Jones and Ram, 2012; Lassalle and Scott, 2018), we discuss innovation adoption practices in the context of these ethnic minority businesses. We are thus exploring how – in times of crisis – forms of innovation can be, indeed must be, embraced (Rogers, 2003) for the sustainability of small businesses (Quinn et al., 2013); even among actors and within communities who have, seemingly, traditionally been reluctant to embrace new innovation, perhaps due to deep-rooted cultural views, but also due to lack of access to relevant sources of finance for their development (Smallbone et al., 2003). The purpose of this paper is thus to empirically explore how a need to innovate was articulated and responded to by Bangladeshi curry restaurant owners, located in the Midlands area of the UK.

To date, discussions about innovation adoption have tended to focus on high-growth operations (Papaoikonomou et al., 2012). Here, we offer a different insight into the nature of innovation, drawing attention to the less glamorous but still significant forms of innovation that were available to ethnic minority entrepreneurs during the pandemic, who had to exploit somewhat mundane and more simple resources to secure their survival and longer-term futures. We further contribute to the extant literature on ethnic minority entrepreneurship by diversifying the way we understand the nature and forms that innovation takes place for contemporary ethnic minority entrepreneurs, particularly in times of crisis. We pay particular attention to the fact that ethnic minority entrepreneurs are acting at the ‘everyday’ (Welter et al., 2017), more localised level of venture creation (Kalantaridis et al., 2019) and are strongly embedded within community structures and local ethnic niches (Lassalle and McElwee, 2016). They often operate within a particular cultural context that sees traditional cultural views and the more modern need to innovate intersect.

Hence, we present the way ethnic minority entrepreneurs re-organise and utilise resources in times of crisis, in ways that are significant, but which have been overlooked in extant literature. We therefore discuss innovation as a response to crisis that can be theorised as being situated between a forced bricolage and a more proactive approach to innovation, leading to diversification.

Our article unfolds as follows. In the next section, we provide an overview of the current literature around entrepreneurship, innovation and crisis; and also consider the particular cultural context our study has to be aware of, owing to its focus on ethnicity. This is followed by a methodology section, in which we explain how we captured and analysed data. We then present our research findings, showing how forms of innovation were adopted within the particular context the pandemic created. A discussion follows, which contextualises our findings in relation to existing literature. Finally, our conclusion considers how future research can expand our contribution.

## Entrepreneurship, crisis and COVID-19 pandemic

Various studies have emphasised the necessity of innovation during crises for firms’ survival, recovery and for competitiveness, especially in the context of small firms (Cefis and Marsili, 2006; 2019; Devece et al., 2016; Quinn et al., 2013). Innovation plays a significant role in influencing and shaping the survival of businesses by providing successful niche strategies, improving existing capabilities and optimization of resources (Cefis and Marsili, 2006). Even before COVID-19, the concept of entrepreneurial crisis has been gaining scholarly attention as a critical part of the entrepreneurial process, and as a way to survive crisis.

Doern et al. (2019) discuss the relationship between entrepreneurship and crisis, showing that entrepreneurship in the context of crisis is not ‘business as usual’. They establish that how entrepreneurs respond to crisis depends on many factors, including experience, stage of business development and resources at the disposal of the entrepreneur. Papaoikonomou et al. (2012) identify coping strategies for negating crisis. They argue that most small businesses suffer from demand shock as they see a fall in demand for their products and services. Some of the strategies for coping with crisis identified in the literature include asset reduction, cost reduction and revenue generation (Hofer, 1980); development and introduction of new pricing models (Reaves and Deimler, 2009); partnership and formation of strategic alliances and marketing and technology innovation (Marino et al., 2008).

Innovation matters especially for populations of entrepreneurs who face challenges to accessing wider formal sources of finance (Quinn et al., 2013), including entrepreneurs from ethnic minority groups (Smallbone et al., 2003). A lack of access to finance, or more generally to support institutions, makes ethnic minority entrepreneurs more vulnerable to crisis, especially and particularly for entrepreneurs who are engaged in labour-intensive sectors, such as catering (Rahman et al., 2018). Historically, in ethnic minority communities, these businesses have often been easier for migrants and ethnic minority entrepreneurs to enter, due to lower financial barriers to entry, and also because the ethnic minority entrepreneurs could rely on their communities for appropriate support (Lassalle, 2018), for example, in terms of supply chains, and to serve as their initial market (Aldrich and Waldinger, 1985). In the past, ethnic minority entrepreneurs have relied on long hours and family labour to establish themselves (Buettner, 2008; McPherson, 2017; Ram et al., 2001), with the unintended consequence of having little opportunities for sustained longer-term growth (Hack-Polay et al., 2020; Rahman et al., 2018), unless they engage into diversification activities in terms of attracting a wider customer base or by diversifying their product and service offering (Lassalle et al., 2021).

The combined and intertwined processes of economic globalisation and human geographic mobility have created opportunities in food culture (Buettner, 2008; Palat, 2015) for migrant and ethnic minority entrepreneurs to propose novel, innovative food experiences and ethnic products to populations in local economies (Chand and Ghorbani, 2011; Lassalle et al., 2021; Zhou, 2004). In this respect, restaurants have adapted ‘curry’ and other traditional South Asian dishes to the UK customer taste and context – sometimes appropriated by other entrepreneurs operating in the UK – demonstrating particular opportunity recognition and exploitation processes among ethnic minority and migrant entrepreneurs (Varman, 2017).

## Ethnic minority entrepreneurship, innovation and technology

Because we focus on how the adoption of innovation and technology becomes a survival strategy for British ethnic minority restaurant owners for surviving COVID-19, our study needs to be aware of the broader cultural context under which ethnic minority entrepreneurship and innovation adoption occur.

Aldrich and Waldinger (1990) suggest that ethnic minority entrepreneurs would rather imitate than innovate. They argue that ethnic minority firms adapt to resources within their environment in their attempt at innovation. They state that ‘rather than breaking new ground in products, process, or administrative form, most businesses simply replicate and reproduce old forms’ (112). Altinay (2010) argues that while ethnic minority entrepreneurs are aware of the benefits of technology and innovation adoption, they are hindered by the perceived barriers to adoption. Furthermore, ethnic minority restaurant owners might be less inclined to view innovation and technology as an integral factor to their business, unlike their mainstream counterparts (Ensign and Robinson, 2011). Nevertheless, second-generation ethnic minority business owners are more likely to be receptive to various innovative behaviour, including on the adoption of ICT, than their first-generation counterparts (Beckinsale and Ram, 2006). For example, Bates and Robb (2013) suggest that the barriers of limited financial resources and expertise – but also the reliance on bonding social capital (Deakins et al., 2007) – constrain the adoption of innovation and technology among older ethnic minority entrepreneurs. Immersion in a host community appears to give immigrant entrepreneurs a proclivity to embrace innovation.

Smallbone et al. (2012) have highlighted the importance of innovation to the success strategies of ethnic minority firms. They identify the many constraints ethnic minority firms face in entrepreneurship. Importantly, they show how some of these constraints are remedied. Likewise, Qureshi and York (2008) identify resource constraints as one of the main reasons for the slow adoption of technology among ethnic minority businesses. Among the many factors influencing the adoption of innovation and technology among ethnic minority firms include lack of top management engagement, knowledge barriers and staff resistance, lack of practical value and other personal incentives, the symbolic value of information technology, poor organisation, poor infrastructure and different concept of time. However, further studies have moved beyond causal factors, to establish how the adoption of technology is a business solution and a strategic site for business support and engagement (Beckinsale et al., 2010).

By focusing on ethnic minority-owned businesses in the hospitality industry, Altinay (2010) stresses the economic and socio-cultural contribution of ethnic minorities to the hospitality industry. She identifies that ethnicity and ethnic affiliation create an environment of emotional and cultural ties between ethnic minority entrepreneurs and their co-ethnic customers. This results in a strong sociocultural environment, which may threaten the adoption of innovation and creativity within the industry. Beckinsale et al. (2010) observe that ethnicity and cultural influences such as involvement in co-ethnic networks hinder the adoption of innovation and technology. Indeed, the literature established that ethnicity, social networks and embedded cultural value and beliefs are significant factors in the adoption of innovation for ethnic minority firms (Chaudhry and Crick, 2004). Mainstream literature in entrepreneurship tends to view ethnic minority firms as lacking innovation, which hinders them from attracting mainstream customers beyond the ethnic community and thus hinders the wider growth of ethnic businesses (Korede, 2021; Strüder, 2003). Yet, Lounsbury et al. (2019) show how culture shapes entrepreneurial innovation and practice. They depart from a reductionist view of culture and ethnicity as constraining entrepreneurial innovation, to show how culture and ethnic affiliations contribute to innovation and novelty.

Against the backdrop outlined above, where polemic exists about how innovative ethnic minority entrepreneurs are, and in mind of the particular challenges and context created because of COVID-19, we provide an empirical insight into restaurant owners’ qualitative experiences of the pandemic, and how they responded to the crisis through particular *forms* of innovation. By so doing, we emphasise how, in times of crisis, forms of innovation *must be,* embraced (Rogers, 2003); even among actors and within communities who have, seemingly, traditionally been reluctant to embrace innovation for different cultural and contextual reasons (Hack-Polay et al., 2020). We now outline the data capturing and analytic processes that inform our contribution.

## Methodology

We conducted semi-structured interviews between May and June 2020, as the UK emerged from the first national lockdown and restaurants were re-opening. At this point, restaurants offered takeaways to their customers. All interviewees were Bangladeshi restaurant owners who have been operating businesses in the UK restaurant industry for between 10 and 24 years. All participants were born in Bangladesh and immigrated to the UK.

Interviewees were recruited through a purposive sampling strategy, which ‘involves identification and selection of individuals or groups of individuals that are proficient and well-informed with a phenomenon of interest’ (Etikan et al., 2016: 2). Interviewees were recruited through a Bangladeshi restaurant owner network in the Midlands of the UK and through the researchers’ informal networks. The respondents were all men, which reflect the current state of the sector in the community.

Semi-structured interviews are flexible means of collecting qualitative data (Kvale and Brinkmann, 2009). We utilised them to capture the general experiences of interviewees as they responded to the uncertain and difficult contexts they encountered, and – more specifically to uncover the forms of innovation adopted. Interviews were conducted online through Zoom – a video conferencing communication platform. In total, 15 restaurant owners were interviewed. Interviews were designed to capture data in interviewees’ own words, revealing how the pandemic created a need for them to innovate, and the forms of innovation – if any – they embraced. In addition, specific questions were asked about interviewees’ use of social media, and ordering and delivery services such as Just Eat, Uber Eats and Deliveroo, to better understand how technology influenced innovation. All interviews were conducted in English, digitally recorded and manually transcribed by the researchers. Table 1 summarises the profile of interviewees.

**Table 1.** Profile of participants.

Names	Location of business	Age	No of restaurants	Years of ownership
Mohammad	Small Heath, Birmingham	40–45	1	11
Ahmed	Stratford-upon-Avon	50–55	2	13
Ali	Walsall	50–55	2	10
Waheed	Small Heath, Birmingham	45–50	2	10
Hassan	Kinver, Stourbridge	40–50	2	19
Mahmoud	Streetly, Sutton Coldfield	45–50	2	24
Rashid	Redditch	35–40	1	18
Asif	Leicester	50–55	1	17
Hussain	Birmingham	40–45	2	10
Khaled	Birmingham	55–60	4	17
Malik	Small Heath, Birmingham	55–60	1	12
Sajid	Walsall	45–50	1	19
Alim	Birmingham	50–55	1	11
Ismael	Stirchley, Birmingham	55–60	1	10
Aminul	Stourbridge	45–50	1	13

### Data analysis

During data analysis, each interview transcript was read and thematically analysed. ‘Thematic analysis is a method for identifying, analysing and reporting patterns (themes) within data’ (Braun and Clarke, 2006: 79). At this stage, we employed an inductive approach to data analysis based on the Gioia method of theory development. Analysis was led by the data to derive conceptual categories (Gioia et al., 2013). During our initial thematic analysis, we used open coding to analyse each transcript, generating first-order codes. In the process, we focused on the experiences of interviewees during the pandemic and the different ‘innovative’ practices they employed. We then organised the codes and removed codes that were redundant. To develop second-order themes, we engaged in repeated comparison of the first-order codes, as the next step of the theorising process (Gioia et al., 2013). This allowed pattern-finding and allowed us to identify and group similar codes, organising them into themes. We further distilled the second-order themes into aggregate dimensions. Three aggregate dimensions were used to categorise the most salient data we captured in terms of revealing the forms of innovation adopted in response to the crisis. These themes are; *struggle and challenges, innovate to survive and survival innovation practices*. Table 2 presents the data structure following the Gioia method. We now discursively present data we grouped in these themes, to answer our research question.

**Table 2.** Data structure.

First-order codes	Second-order themes	Aggregate dimensions
Loss of income and livelihood	Loss and threat	Struggles and challenges
Loss of valuable resources, employees etc.		
Pandemic threatens business survival		
Cultural side of being a man and having problems	Cultural dilemmas	
Pandemic threatens long-held cultural practices within the industry		
Feeling of cultural alienation from work		
Lockdown spent as a time to reflect on faith and pray	Religion and rituals	
Ramadan and religious practices		
Adoption of methods and practices they would not have pursued	Bricolage	Innovate to survive
Conversion and redeployment of resources		
Finding new uses for existing and redundant resources		
Changed from an eat-in model to a takeaway model	Change in business model	
Social distance and spatial constraints threaten existing business model		
Prioritize cashless payment	Payment solutions	Survival innovation practices
Increasing adoption of ePOS system		
Integration of payment system on their websites		
Use of social media marketing through Twitter, Facebook, Instagram etc.	Active usage of social media and food delivery apps	
Adoption of online food ordering services such as Just Eat, Uber Eats etc.		
Private demand for chefs	Home catering	
Home services and deployment of chef to homes		
Operation of afternoon service	Introduction of business lunch menus	
Lunchtime menus		

## Findings

**Figure 1.**
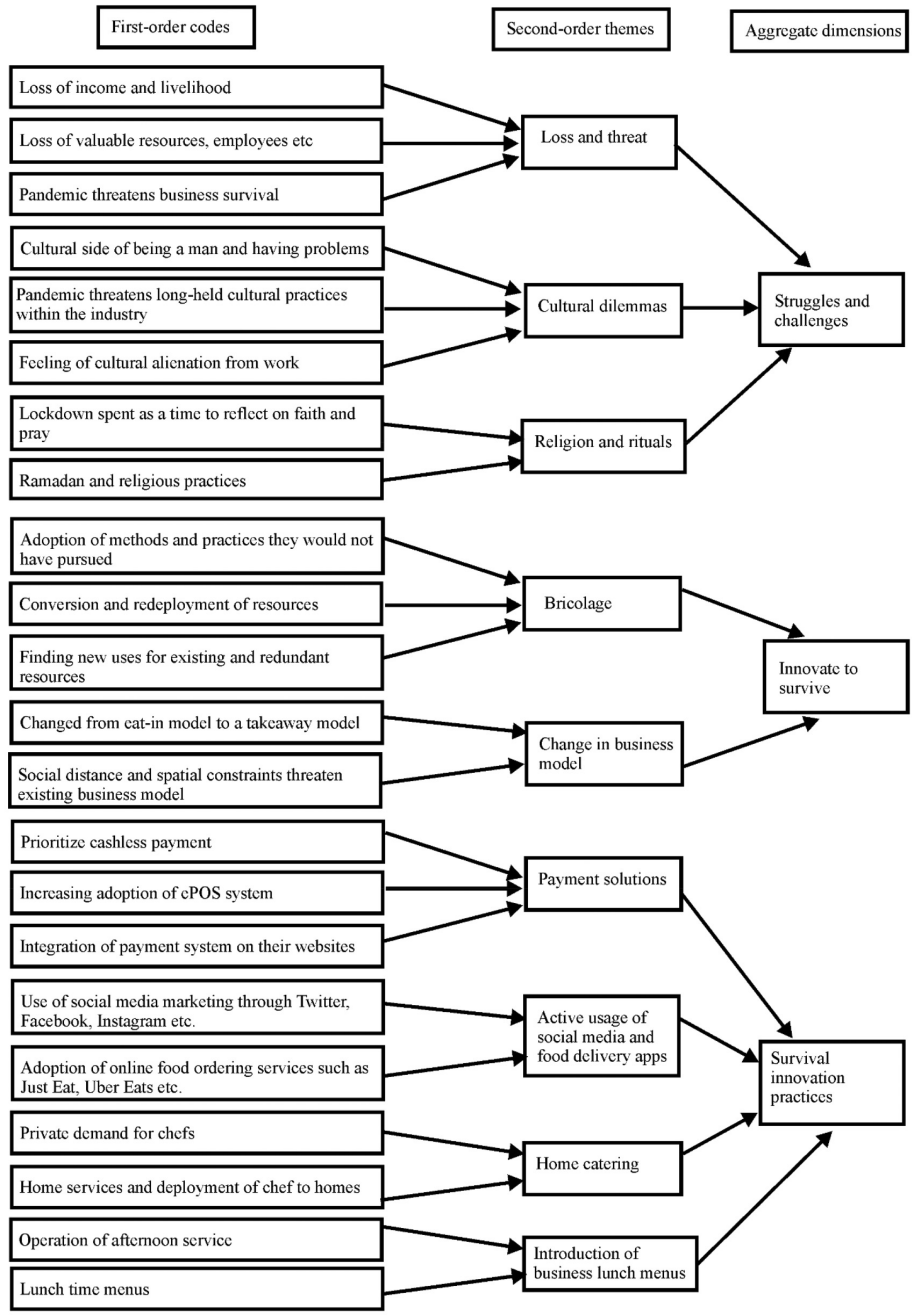
Data visualisation.

Our findings reveal the struggles and challenges ethnic minority entrepreneurs experienced during the first national lockdown due to the COVID-19 crisis. Thereafter, we present their reactive approach to the crisis. Specifically, COVID-19 creates a context where entrepreneurs considered innovation as the solution for survival. Our findings then reveal the major innovation practices entrepreneurs used to negotiate survival during the crisis. Figure 1 presents a visualisation of our findings which, in line with the Gioia method, is grounded in the codes that our research process derived.

## Struggles and challenges

### Loss and threats

The pandemic created profound challenges to ethnic minority entrepreneurs operating in the restaurant industry; linked especially to feelings of loss and threats of business and livelihood. In turn, anxiety about the future was experienced. *Ali* compared the crisis to a ‘big rock falling on his head’, demonstrating a sense of losing momentum, stating:

I have been working really hard to build my business for the last three years and I have started to see the improvement. This pandemic has ruined all my plans, it's like a big rock just fell on top of my head.

Similarly, *Mahmoud* commented:

I have never closed the restaurant for a single day in my 27-years working life, and suddenly it was shut for an unknown period. I didn’t know what to do. I was lost.

Further experiences of loss we captured related to a loss of income and the loss of trusted staff members, who cannot be furloughed. Interview data suggested a strong rapport between restaurant owners and employees, who are often seen as ‘family members’. This rapport may be based on shared ethnicity. Having to lose employees in this context caused strong emotional as well as financial challenges.

### Cultural dilemmas

The pandemic created cultural challenges and ambivalences, seeing long-held established tradition norms and beliefs clashing with the contemporary need to innovate, modernise and ‘westernise’ through technology. For example, *Asif* was torn between adopting delivery services and ‘holding on’ to the cultural practice of greeting and interacting with customers face-to-face:

I don’t believe in delivery platforms (such as using Deliveroo and Just Eat), because you don’t get to see your customers, you don’t get to say hi and hello. You only communicate on the phone or online. So there is no personal touch there. I would rather see my customers, greet them, engage with them and interact with them. But with these delivery services, you don’t get to interact with your customers … I would rather see my customer and build customer relationship.

Another cultural dilemma experienced relates to being forced to ‘stay at home without work’. The men expressed how inherent and interwoven their gendered identities are to their working practices. The men expressed patriarchal and ‘bread winner’ mentalities (see also Bruni et al., 2004): ‘Men are supposed to go to work and not hang around the house’. Culturally, the pandemic challenged the long-held associations between work and masculinity. *Hassan* stated:

We’re not designed for staying home for 24 hours. I’m very passionate to come to work. I love my job. My happiness is going to work, meeting and greeting people, you know, and COVID-19 has taken that away from me.

In the most extreme case, *Malik* saw the forced hiatus as shameful and profoundly emotionally challenging:

I didn’t know what to do and felt helpless. I was crying silently. I was ashamed of crying out loud.

### Rituals and religion

The majority of the Bangladeshi restaurant owners are Muslim. An unexpected relationship between enterprise, religiosity and the pandemic was articulated. Enterprise demands long hours and stress. In turn, many interviewees suggested a general sense of being disconnected from their faith had defined their enterprise journeys pre-pandemic. However, the enforced hiatus from enterprise created the opportunity for some to ‘reconnect’ with their faith. Ethnic minority entrepreneurs found cathartic solace and support in their faith and teachings of Allah. Indeed, the pandemic was seen as ‘the will’ and ‘permission’ of Allah, and therefore something they had to accept at a spiritual level. This perhaps meant interviewees experienced the pandemic with a higher level of acceptance and spiritual meaning. Ramadan occurred during the lockdown. This gave entrepreneurs further time and context to reflect on their faith, and fast *Ismael* commented:

I use the time to perform namaz more, read the Quran more and get closer to Allah. For the first time in my working life, I actually had so much free time. I didn’t have any excuse not to pick up the Quran. I didn’t have an excuse not to observe my namaz … Ramadan came along as well. It makes me feel better spiritually.

## Innovate to survive

The ethnic minority entrepreneurs we studied identified the need to innovate as a survival mechanism. As the nation began to come out of the first lockdown, and restaurants were about to open again, innovation was perceived as something of a necessity to reach new markets, attract new staff and – in particular – engage and attract younger customers. *Waheed* identified that ‘only innovative’ restaurants will survive the pandemic:

Only innovative approaches will help the ethnic minority-owned restaurants to survive the post-pandemic world, those who will not be able to innovate or change will die out from this pandemic.

We identified two major ways ethnic minority restaurant entrepreneurs innovate during the pandemic: bricolage and change in business model 

### Bricolage

Bricolage refers to finding new uses for existing and redundant resources. This involves the re-organisation and deployment of new and existing resources to survive the pandemic. Existing resources such as staff and smartphones were redeployed to meet changes in business operations. For example, some of the chefs were converted to local delivery drivers. Smartphones and tablets – otherwise underused – were utilised in making videos of menus and posted on social media. One of the entrepreneurs, *Alim,* identified that he used IT savvy staff to develop social media marketing, offering discount to customers via online vouchers:

We have converted some of the younger and capable staff who are good with technology and social media to start creating and posting online contents … they post new menu in online platforms and offers discounts to people.

Likewise, *Sajid* noted how he has introduced business lunch and redeployed staff to deliver lunch to local businesses, overcoming concerns customers have about the spread of the COVID-19. He stated:

What we have started doing is to look for new ways of serving our customers … For example, we have started a business lunch service, targeting some businesses nearby and delivering menu to them during lunchtime.

### Change in business model

The traditional business model in most ethnic minority restaurants has been defined by relatively low costs for customers and a ‘eat in’ approach: the idea that curry should be ‘cheap and cheerful’, enjoyed in a social atmosphere among friends and families. *Ahmed* described it as:

A group of friends going out for a lively evening of entertainment finished off with a curry is part and parcel of the British way of life. If you take that away you’re taking away the very essence of what going out for a curry means.

However, the pandemic and the associated social distance and lockdown threaten this business model. The government's policy of 2 metres social distancing requirements means that restaurants cannot accommodate the number of customers they once did due to spatial constraints. In turn, the model has shifted from an eat-in model to a takeaway model. This is illustrated by the narrative of *Mohammad* below:

My restaurant was mainly providing eat-in service and no delivery service. However, I had to think about alternative ways to keep my business going during the pandemic and lockdown. I am now mainly surviving on takeaway service and contactless delivery service. It's nowhere near the previous trade, but it is keeping me going for now.

In many cases, this switch has helped restaurants to remain partially open and survive the loss of income.

## Survival innovation practices

Here, we focus on the major innovation practices entrepreneurs used to negotiate survival during the pandemic. These innovative practices emanate from challenges, crises and constraints experience during the pandemic. We found that COVID-19 is changing the business behaviours and practices of ethnic minority restaurant entrepreneurs, causing them to reconsider old and traditional business practices.

### Payment solutions

The COVID-19 pandemic and the associated social distancing have pushed ethnic minority restaurant owners to transition from a predominant reliance on cash payments to a ‘cashless’, card-payment format. In response, ethnic minority restaurant owners have integrated payment systems on their websites (e.g. the ePOS platform). Although some of the restaurant owners identify that such integration is expensive, they see it as a necessary investment for the changing landscape they are operating in. As put by *Malik*:

What I am doing is that I am getting my own system. I have got my website with PayPal but I am getting my ePOS system too. I have contacted the website company, and work is going on to deliver it soon … It is an investment I am making to secure the future of the business.

### Active usage of social media and food delivery apps

As with payment methods, the need to utilise technology in relation to food delivery was expressed; specifically, social media and apps. This utilisation was striking because before the pandemic, most interviewees have not used social media or food delivery apps. Indeed, some were mistrusting of technology, and believed the substitution of face-to-face interaction through technology could only be a bad thing. The cost of technology was a further perceived barrier. The below illustrates the adverse view of food deliver apps held by some participants before the pandemic:

No, I don’t believe in them (delivery platforms). I don’t believe in giving my business to those people when I can do it myself. I don’t want my profit going to companies like Just Eat. They found a loophole where they can make money from all these restaurants. If I can do it myself, while should I give my business away to other people (*Hassan*)

So rather than paying them percentages, I’ll rather give that percentage to my customers. The profit margin in our industry is very little and people don’t want to pay too much for the curry (*Rashid*)

All the hard work is done by our little restaurants and Just Eat come and sweep away the money from under our feet (*Sajid*)

However, an embracement rather than mistrust of social media and food deliver apps now exists. For example, *Aminu* commented:

My main business is based on our community and my customers come to eat in so I had little need for technology and IT … but now, people are at home and there is social distancing … We now use staff and family to do social marketing. We post pictures of the menu and videos now to engage with customers.

A further feature of this finding relates to owners relying on younger, more technological savvy, family members to manage the integration of technology in their enterprises, for example, sons and daughters updating social media accounts and marketing campaigns. In this way, some of the fears associated with technology were dissipated. *Khaled* commented:

We have been slow to adopt technology because we didn’t create the industry, it was passed on by our older generation … my son is good with technology and supports the business with social media posting … we need to attract the younger generation, they are willing to learn, they are more energetic and more creative, they can take the business to a new level.

### Home catering

Some restaurant owners sent chefs and cooks to people's homes to cater. *Mohammed* identified a ruse in demand for home catering in the immediate weeks after the first lockdown was lifted:

Before now, we used to do outside catering, covering community events, weddings, birthday parties. I lost all my business because of the lockdown and all my pre-booked outside catering events have been cancelled. But now, I am sending chefs to peoples’ houses to cook and cater for small house parties and events for about 20 people … this is a new additional service to the business.

### Introduction of business lunch menus

Further, four of the respondents introduced business lunch menus. This is an important finding because traditionally, ethnic restaurants do not operate afternoon services. Besides, ‘ethnic restaurants have been increasingly vulnerable to competition from other forms of eating out’ (Ojo, 2018: 36); are now competing for eat-out customers. *Sajid* noted how he has introduced business lunch delivery service to local businesses to overcome the concerns customers have about the spread of the COVID-19. He stated:

What we have started doing is to look for new ways of serving our customers … For example, we have started a business lunch service, targeting some businesses nearby and delivering menu to them during lunchtime.

Having presented empirical data, we now discuss our study, positioning it and contextualising its theoretical contributions.

## Discussion

Findings show how ethnic minority restaurant owners in the UK have adopted diverse innovative practices as a solution for survival. Identified forms of entrepreneurial bricolage in the context of ethnic minority businesses make contributions to debates in the literature. First, by bringing the idea of resilience in the innovation narrative, it complements research on *forms of diversification* in the ethnic minority entrepreneurship literature (Lassalle and Scott, 2018; Ram et al., 2001). Specific to ethnic minority entrepreneurs, innovation is also considered as part of the first and second-generation discussions (McPherson, 2017); diversifying the way literature has tended to see first and second-generation immigrants as distinctive in their ability and willingness to innovate. Second, it builds on the notion of early adopters (Rogers, 2003) to make a distinction between resistant and proactive entrepreneurs in terms of innovation. This debate, whilst well-rehearsed in other fields (Bessant et al., 2015; Cefis and Marsili, 2019; Devece et al., 2016), brings a novel understanding to the specific study of ethnic minority entrepreneurship and the contexts it operates in. We further this debate with reference to crisis, which – we show – acts as a triggering event for innovation, whereby ethnic minority entrepreneurs find their own solutions when existing support is insufficient, and are willing to reposition long-held cultural views to ensure innovation adopted succeeds.

This offers a fresh perspective, by emphasising not only how innovation improves the competitive strategy of firms and increase profitability in a neo-classical sense (Cefis and Marsili, 2019), but also by revealing how seeking new ways of managing resources and the uptake of technologies (which were previously considered unnecessary) to survive external induced crisis beyond the control of the entrepreneur. It therefore engages with considerations of the *sustainability* of ethnic minority business, through diversified forms of income generation (Lassalle and Scott, 2018). It acknowledges that most of ethnic minority entrepreneurs in the restaurant industry in the UK are indeed middlemen serving customers with ethnic goods (Lassalle et al., 2021). As studied elsewhere (Palat, 2015; Ram et al., 2001), they have a limited market to serve and strongly rely on long hours to sustain their activities, even outside of crisis. In the past, this ‘model’ and the long hours it demanded somewhat transcended the need to innovate; but the context of COVID-19 changes that, and saw innovation embraced accordingly.

Our analysis shows that while crises often constrain innovation in small firms (Smallbone et al., 2012), crises may also trigger the adoption of innovations that are necessary for the survival of firms. Some of our respondents suggest that innovation is an ‘emergency’ response that is critical for business survival during a crisis. Survival innovation is not geared towards growth, it is aimed at sustenance and survival (Villares-Varela et al., 2018); especially, when businesses face disruption to their operations and activities due to external crises beyond the control of the entrepreneur, as it is the case with the current pandemic. The adoption of innovation for survival purposes demonstrates that innovations do not have to be new to be effective, but existing innovations are exploited and deployed *in new ways* when the survival of the business is threatened. Such innovation is necessary to ethnic minority entrepreneurs for surviving crisis, especially among entrepreneurs operating at the margins of the economy, in labour-intensive sectors (Ishaq et al., 2010; Ram et al., 2001). We contend that crisis such as COVID-19 offers researchers opportunities to think differently about the conception of innovation; revealing how innovation can be conceived as finding new uses for existing capabilities and resources, and how existing technologies take on new meanings during crises. We argue that this mundane nature of innovation, a form of entrepreneurial bricolage (Baker and Nelson, 2005; Villares-Varela et al., 2018; Simba et al., 2021) is critical to surviving crises and overcoming entrepreneurial challenges during crises for the vast majority of enterprises operating in the UK, especially ethnically owned enterprises. However, although the desire for business survival is driving the uptake of innovation and technology, the adoption of innovative technology itself does not guarantee the survival of these restaurants in the long term. Indeed, many of the respondents observe that a good number of curry houses will be out of business due to the pandemic, irrespective of whether they adopt innovation or not. However, the adoption of innovation is likely to improve their chance of survival (Quinn et al., 2013). It is a necessity to survive, but not in itself something that makes survival inevitable.

This aligns well with debates on diversification and bricolage in the literature interested in the strategies and practices of migrant and ethnic minority entrepreneurs. This literature emphasises the need for ethnic minority businesses to diversify their activities to capture a larger market base, therefore extending beyond the boundaries of the (ethnic) enclave economy (Lassalle and Scott, 2018; Zhou, 2004). We observe that in such contexts of crisis, the incentive to break-out has been intensified. The pandemic has threatened the survival of the majority of the businesses, as the hospitality industry is significantly hit by the national lockdown. Restaurant entrepreneurs are running out of funds, as the government support is barely enough to cover minimal cost Further, the literature often suggests that diversification is most often achieved by the second generation of ethnic minority owners. Our results show that whilst the new generation indeed provides the skills and knowledge to diversify, the emergency of the crisis encourages an earlier adoption of change, including on the technological basis.

## Adoption of innovation among ethnic minority entrepreneurs

COVID-19, and the associated social distancing measures implemented, bring challenges to ethnic minority entrepreneurs operating in the restaurant sector. For instance, the number of customers that could be served is reduced, when the restaurant can be opened at all. The COVID-19 crisis is triggering the adoption of innovative technologies, as ethnic minority entrepreneurs prioritize business survival over, reversing existing cultural barriers and the lower rate of low adoption of innovation among ethnic minority firms (Beckinsale et al., 2010). While cultural nuances continue to affect the uptake of innovation, this study shows that a crisis may force and enable ethnic minority firms to adopt technology when their survival is threatened. Despite having been sustainable in business for many years prior to the pandemic, for these entrepreneurs, innovation becomes a necessity. They are willing to jettison some more traditional ways of doing business to explore alternative and innovative routes for survival, therefore using other resources (including younger generation's knowledge) to diversify their activities. COVID-19 thus becomes an important push factor and an enabler of adoption of innovation in the ethnic minority restaurant industry. However, individual ethnic minority entrepreneurs have responded differently to this push, adopting different attitudes towards innovation; some engaging in small adaptative steps, and others really exploring innovative practices. As shown in the findings, various practices have been implemented from novel payment solutions as a response to long-established customers’ requests, new usage of social media and food delivery apps, or the offering of new services, such as home catering. In more theoretical terms, ethnic minority entrepreneurs have engaged in different forms of innovation (Bessant et al., 2015; Rogers, 2003), transitioning from more resistant to more proactive approaches, triggered by the crisis. Their approaches also have been diverse, whether the adoption of innovation and technology is triggered by the changing needs of the customer, or to reach more mainstream customers, as one of the phases of the diversification process (Lassalle and Scott, 2018). Apart from reaching new and mainstream customers (and therefore increase the customer base beyond the community market), ethnic minority entrepreneurs also want to attract the younger generation within the ethnic community who are technology savvy. Such customer-centric pushes to adopt innovative practices are driven both by the crisis itself and by changing customers’ preferences to the entrepreneurial offerings of ethnic minority entrepreneurs. Ethnic minority entrepreneurs also build on the recommendations from within their community, including from other entrepreneurs from the same ethnic minority community. In such a case, the adoption is based on the imitation of best practices within the Bangladeshi community of restaurant owners. Such a form of slow adoption (Rogers, 2003) from ethnic minority networks relies on the connections and networks within the community. It also optimizes the usage of scarce resources by relying on such informational support sought within the community networks.

## Between bricolage and proactive approaches to innovation in ethnic minority entrepreneurship

In the context of ethnic minority entrepreneurship, innovation as a response to crisis can be theorised as being situated between a forced bricolage (Desa and Basu, 2013; Villares-Varela et al., 2018) and a more proactive approach to innovation, leading to diversification (Lassalle and Scott, 2018). In both cases, resources are limited by the nature of the industry, lack of capital access and by the limited amount of institutional support available.

However, we see that through engagement with customers (and their changing needs) and with intergenerational ethnic community networks, including with more technology-savvy individuals, ethnic minority entrepreneurs are able to respond to customers’ needs and by that to diversify their activities and their customer base through innovation. They are able to prioritise and re-organise resources ‘at hand’ (Lassalle et al., 2020) to survive external induced crises beyond the control of the entrepreneur, such as Brexit (Hall et al., 2020), financial crisis (Santos et al., 2021; Smallbone et al., 2012) or the COVID-19 pandemic. These events affect the activities (and the lives) of ethnic minority or migrant entrepreneurs. In fact, we can argue that through such mundane and serendipitous decision-making (Lassalle, 2018) leading to the adoption of innovative practices and technology, the crisis changed ethnic minority entrepreneurs’ ability to recognize novel opportunities in their local markets (Kloosterman, 2010). They have converted threat (Just Eat, Deliveroo, etc.) into opportunities to gain access to a broader, younger clientele, but also to have greater access to an increasingly mainstream customer base. Crisis, as an enabler of innovation (Rogers, 2003) has turned innovation resistant ethnic minority entrepreneurs to innovation adopters. Conceptually, it shows that although ethnic minority businesses, as many other small firms are vulnerable, they can demonstrate their ability to adapt and innovate as a response.

## Conclusion

This article has explored the adoption of innovation by ethnic minority entrepreneurs during a time of crisis (COVID-19). The findings show that within an industry that is slow to innovate due to cultural norms and practices, the pandemic has caused many restaurant owners to rethink their business models and embrace innovation – often without reluctance – to heighten their chances of survival in the context created by the pandemic. We show that while ethnic minority businesses, as many other small firms are vulnerable to the pandemic and crisis more generally, they are reflexive and able to adapt and innovate as a response.

Participants in this study are all male entrepreneurs. Whether innovation practices vary between males and females is not something our research investigates, but provides a relevant question to address in the future to further contribute to knowledge of women migrant entrepreneur's experiences and specific challenges in different contexts (e.g. Lassalle and Shaw, 2021; Strüder, 2003). Our research faces limitations common to many qualitative works interested in the experiences of specific communities. Nevertheless, in-depth data collection and rigorous coding (following Gioia et al., 2013) enable theorisation, if not generalisability of the findings. We are also aware that ethnic minority entrepreneurs operating in the restaurant industry were still in the middle of the crisis during the time of the fieldwork. Whilst this might limit the reflective nature of the interviews, it can also generate more insight into the actual practices (Thompson et al., 2020). Finally, we acknowledge that our fieldwork is limited to a particular geographical context and we would encourage further studies in different locations, including outside of the UK for further insight.

We encourage other researchers to look at the nuanced ways entrepreneurs innovate in response to crisis, including with the current pandemic and other socio-political phenomenon (e.g. the forthcoming, ongoing impact of Brexit). During economic crises, the need to innovate is amplified. As the UK moves into a post-Brexit, post-COVID-19 future, it is likely that new forms of innovation and adaption will be displayed among ethnic minorities and other everyday entrepreneurs (Welter et al., 2017) as a reaction to the changing conditions in which they operate. This gives scholars the opportunity to longitudinally examine the forms of innovation adopted within a changing socio-economic landscape. While crisis may seem overwhelming, practically it forces innovation and thus encourages innovation; making crisis a valuable context to explore innovation within. For practitioners working in support of ethnic minority entrepreneurs and for entrepreneurs themselves, the paper reveals that in such difficult conditions, the adoption of innovation through specific activities should be considered even though they could be counter-intuitive for the entrepreneur. The adoption of innovation as this level of everyday and mundane practices (rather than a neo-liberal discourse on innovation) can help the businesses not only to survive but to actually engage in diversification and further developments.
